# Impact of a restrictive antibiotic policy on the acquisition of extended-spectrum beta-lactamase-producing Enterobacteriaceae in an endemic region: a before-and-after, propensity-matched cohort study in a Caribbean intensive care unit

**DOI:** 10.1186/s13054-021-03660-z

**Published:** 2021-07-26

**Authors:** Christophe Le Terrier, Marco Vinetti, Paul Bonjean, Régine Richard, Bruno Jarrige, Bertrand Pons, Benjamin Madeux, Pascale Piednoir, Fanny Ardisson, Elain Elie, Frédéric Martino, Marc Valette, Edouard Ollier, Sébastien Breurec, Michel Carles, Guillaume Thiéry

**Affiliations:** 1 Division of Intensive Care, University Hospital of Guadeloupe, Pointe-à-Pitre, Les Abymes, French West Indies France; 2 Division of Intensive Care, Geneva University Hospitals, 4 Rue Gabrielle-Perret-Gentil, 1211 Geneva 14, Switzerland; 3 Division of Intensive Care, Saint-Pierre Clinic, Ottignies, Belgium; 4 Division of Clinical Epidemiology, University Hospital of Saint-Etienne, Saint-Etienne, France; 5 Division of Hospital Infection Control, University Hospital of Guadeloupe, Pointe-à-Pitre, Les Abymes, French West Indies France; 6 Laboratory of Clinical Microbiology, University Hospital of Guadeloupe, Pointe-à-Pitre, Les Abymes, French West Indies France; 7 Faculty of Medecine Hyacinthe Bastaraud, University of Antilles, Pointe-à-Pitre, French West Indies France; 8 INSERM Center for Clinical Investigation 1424, Pointe-à-Pitre, Les Abymes, French West Indies France; 9 Transmission, Reservoir and Diversity of Pathogens Unit, Institut Pasteur de Guadeloupe, Pointe-à-Pitre, French West Indies France; 10 Division of Intensive Care, University Hospital of Saint-Etienne, Avenue Albert Raimond, 42270 Saint-Priest-en-Jarez, France; 11 University Jean Monnet, Saint-Etienne, France

**Keywords:** Intensive care unit, Caribbean, Extended-spectrum beta-lactamase (ESBL)-producing *Enterobacteriaceae*, Antimicrobial resistance, Antibiotic stewardship, Intestinal microbiota, ESBL-E colonization

## Abstract

**Background:**

High-level antibiotic consumption plays a critical role in the selection and spread of extended-spectrum beta-lactamase-producing *Enterobacteriaceae* (ESBL-E) in the ICU. Implementation of a stewardship program including a restrictive antibiotic policy was evaluated with respect to ESBL-E acquisition (carriage and infection).

**Methods:**

We implemented a 2-year, before-and-after intervention study including all consecutive adult patients admitted for > 48 h in the medical-surgical 26-bed ICU of Guadeloupe University Hospital (French West Indies). A conventional strategy period (CSP) including a broad-spectrum antibiotic as initial empirical treatment, followed by de-escalation (period before), was compared to a restrictive strategy period (RSP) limiting broad-spectrum antibiotics and shortening their duration. Antibiotic therapy was delayed and initiated only after microbiological identification, except for septic shock, severe acute respiratory distress syndrome and meningitis (period after). A multivariate Cox proportional hazard regression model adjusted on propensity score values was performed. The main outcome was the median time of being ESBL-E-free in the ICU. Secondary outcome included all-cause ICU mortality.

**Results:**

The study included 1541 patients: 738 in the CSP and 803 in the RSP. During the RSP, less patients were treated with antibiotics (46.8% vs. 57.9%; *p* < 0.01), treatment duration was shorter (5 vs. 6 days; *p* < 0.01), and administration of antibiotics targeting anaerobic pathogens significantly decreased (65.3% vs. 33.5%; *p* < 0.01) compared to the CSP. The incidence of ICU-acquired ESBL-E was lower (12.1% vs. 19%; *p* < 0.01) during the RSP. The median time of being ESBL-E-free was 22 days (95% CI 16-NA) in the RSP and 18 days (95% CI 16–21) in the CSP. After propensity score weighting and adjusted analysis, the median time of being ESBL-E-free was independently associated with the RSP (hazard ratio, 0.746 [95% CI 0.575–0.968]; *p* = 0.02, and hazard ratio 0.751 [95% CI 0.578–0.977]; *p* = 0.03, respectively). All-cause ICU mortality was lower in the RSP than in the CSP (22.5% vs. 28.6%; *p* < 0.01).

**Conclusions:**

Implementation of a program including a restrictive antibiotic strategy is feasible and is associated with less ESBL-E acquisition in the ICU without any worsening of patient outcome.

**Supplementary Information:**

The online version contains supplementary material available at 10.1186/s13054-021-03660-z.

## Background

Antibiotic resistance is among the most important public health concerns worldwide [1]. Recently, the World Health Organization published a global priority list of antibiotic-resistant bacteria in which extended-spectrum beta-lactamase-producing *Enterobacteriaceae* (ESBL-E) are included in the priority 1 group [2]. In intensive care units (ICUs), ESBL-E have been increasingly reported for many years, which strengthens the requirement for efficient prevention strategies [3]. Occurrence of ESBL-E in the ICU may result either from the introduction of an exogenous strain through newly hospitalized patients with a possible further dissemination through cross-contamination, or from the in vivo selection of resistant isolates from preexisting strains, mainly in the gut microbiota, through horizontal gene transfer [4]. Among risk factors for ESBL-E acquisition, antibiotic exposure to broad-spectrum cephalosporins and beta-lactam/beta-lactamase inhibitor combinations has been identified as an independent risk factor for colonization or infection with ESBL-E pathogens [5].

Antibiotic therapy is heavily used in ICUs where it has been reported that more than 70% of patients are treated with at least one antibiotic [6]. Consequently, antibiotic overuse and the resulting selection pressure makes the ICU an important determinant of the spread of ESBL-E in the hospital [7, 8]. Different stewardship policies have been developed and implemented in many settings, including ICUs, to improve antibiotic use and clinical outcomes and to reduce the overall antibiotic selective pressure [7–9]. Among the strategies that have been implemented to optimize antibiotic prescription in ICUs, some restrictive policies, such as delaying the initiation of antibiotics in selected patients or avoiding broad-spectrum antibiotic therapy, have been successfully proposed [10].

In the Caribbean region, the prevalence of multidrug-resistant bacteria is high, including ESBL-E [11, 12], and this particular local ecology often leads clinicians to use broad-spectrum antibiotics empirically [13]. Although stewardship programs are urgently needed, no such restrictive strategy has been evaluated in ICUs. In order to overcome this issue, we implemented a stewardship program based on a restrictive antibiotic policy. The aim of this study was to evaluate the impact of this strategy on ESBL-E acquisition in the ICU when compared to a conventional and unrestricted antibiotic policy.

## Methods

### Study design and setting

We conducted a retrospective, observational, before-and-after intervention study from 1 January 2014 through 31 December 2015 in a 26-bed ICU admitting medical and surgical patients at Guadeloupe University Hospital (French West Indies). The ethics committee of the French Society of Intensive Care Medicine (CE SRLF 18-44) approved the study and granted a waiver for informed consent as both treatment methods were classified as standard care. This trial follows the STROBE statement for the reporting of cohort studies.

Rectal swabs (ESBL-E screening) were performed at ICU admission and once-weekly until discharge, as well as upon admission to the next unit. In the latter case, positive ESBL-E carriage was attributed to the ICU. Contact isolation precautions were applied for each patient until the first swab results were obtained. Alcohol-based handrub was routinely used for hand hygiene. None of these procedures was modified during the study period. All patients admitted to the ICU > 48 h during the study period were included in the analysis and followed up until hospital discharge or death. Patients for whom ESBL-E carriage was unknown on ICU admission were not included.

The outcome of interest was the median time of being ESBL-E-free in the ICU, defined by the time to acquire an ESBL-E in a competing event of death during follow-up. Secondary outcomes were the incidence of ICU-acquired ESBL-E, duration of antibiotic therapy, antibiotic-free days until ICU discharge, all-cause hospital and ICU mortality, ICU and hospital length of stay, ICU-acquired infections and bacteremia with ESBL-E and relapse or recurrence of sepsis. Subgroups of ICU patients receiving antibiotic therapy and in septic shock were also analyzed to investigate outcomes in those directly exposed to the restrictive antibiotic stewardship strategy.

### Procedures

The 2-year study period was split into two 1-year periods, which differed by the antibiotic policy employed. During the first year, the “conventional strategy period” (CSP), antibiotic therapy was prescribed at the physician’s discretion based on national and international guidelines. This strategy included the use of a broad-spectrum antibiotic as initial empirical treatment in the case of sepsis or suspected infection, followed by de-escalation after 48 to 72 h, based on microbiological data. The main regimens were combination therapies with a cephalosporin and aminoglycoside for community-acquired infections, and carbapenem or piperacillin/tazobactam combined with amikacin for hospital-acquired infections. Dosage, timing and duration followed French guidelines [14].

As part of a stewardship program, a new set of guidelines with a restrictive antibiotic protocol was established by the ICU team, approved by a multidisciplinary team and implemented on 1 January 2015. The “restrictive strategy period” (RSP) was based on seven principles. (1) For suspected infection, microbiological samples were taken immediately, and antibiotic therapy was initiated only after microbiological identification, except for septic shock, severe acute respiratory distress syndrome (ARDS) and meningitis. (2) For non-documented septic shock and severe ARDS, an empiric combination therapy including a cephalosporin and an aminoglycoside was immediately started after microbiological sampling according to the ICU protocol. Combined therapy included either second or third cephalosporins (cefuroxime, cefotaxime or ceftriaxone) for community-acquired septic shock, or cefoxitin for hospital-acquired septic shock (owing to the resistance to the previously listed cephalosporins and the high rate of susceptibility to cefoxitin of the ESBL-E) or an anti-*Pseudomonas aeruginosa* cephalosporin (ceftazidime or cefepime) for late (> 5 days) ventilation-acquired pneumonia (VAP). The second antibiotic was amikacin, unless a Gram-positive pathogen was highly suspected. Due to the low prevalence of methicillin-resistant *Staphylococcus aureus* in our hospital, the first-line anti-staphylococcal treatment was cefazolin. (3) No use of piperacillin/tazobactam and carbapenems for empirical treatment, only for a documented infection without an alternative option. (4) Limited coverage on *P. aeruginosa*, unless indicated. (5) Limited coverage on subdiaphragmatic anaerobes, unless indicated. (6) Monotherapy as a definitive treatment. (7) Other characteristics of antibiotic treatment were short duration, high doses and de-escalation as soon as possible to the narrowest alternative [15], with a focus on penicillin, first- and second-generation cephalosporins, according to the attending physician and following ICU protocols (see detailed protocol in Additional file 1). The local epidemiology of resistant strains according to the unit and origin of samples in 2014–2015 is provided in Table 1: Additional file 1.

### Data collection

Clinical and laboratory findings were collected from the patient’s medical records. In the CSP, diagnosis of infection and sepsis was based on the clinical judgment of the attending physician. During the RSP, the diagnosis required the identification of a pathogen and/or a source of infection. Septic shock was defined by an infection associated with the need of vasoactive drugs.

ESBL-E carriage on ICU admission was defined by a positive rectal swab or positive culture without evidence of clinical infection. ICU-acquired ESBL-E was defined by a positive swab or culture 48 h or more after a negative culture at admission. ESBL-E infection was defined as a positive culture with evidence of clinical infection and detected by chromID ESBL® (bioMérieux, Marcy l’Etoile, France), a ready-to-use chromogenic selective medium for ESBL-producing *Enterobacteriaceae*. Antibiotic susceptibility was tested by the disk diffusion method on Mueller–Hinton agar (Bio-Rad, Hercules, CA, USA), and production of ESBL was confirmed by the double-disk synergy test according to the guidelines of the European Committee on Antimicrobial Susceptibility Testing [16]. Due to the low incidence in our hospital, carbapenemase-producing *Enterobacteriaceae* were not analyzed in this study. VAP was diagnosed using standard criteria [17]. Diagnosis of VAP required microbiological confirmation by quantitative culture of a common pathogen. Other ICU-acquired infections were diagnosed using standard criteria, with microbiological documentation for all cases.

### Statistical analysis

Data are reported as frequencies and proportions for categorical variables and mean, median, standard deviation and 1st and 3rd quartiles for continuous variables. Patients hospitalized in the CSP were compared with those hospitalized in the RSP using the Chi-square or Fisher’s tests for categorical variables and Wilcoxon–Mann–Whitney tests for continuous variables. To assess the relationship between the strategy period and the median time of being ESBL-E-free, we calculated the Kaplan–Meier curve and used a univariate Cox proportional hazard regression model to estimate the hazard ratio (HR) and its 95% confidence interval. The follow-up time used for survival analyses corresponded to the time between ICU admission and ESBL-E acquisition, or ICU discharge from intensive care (or death) if no ESBL-E acquisition occurred during ICU stay.

**Fig. 1:**
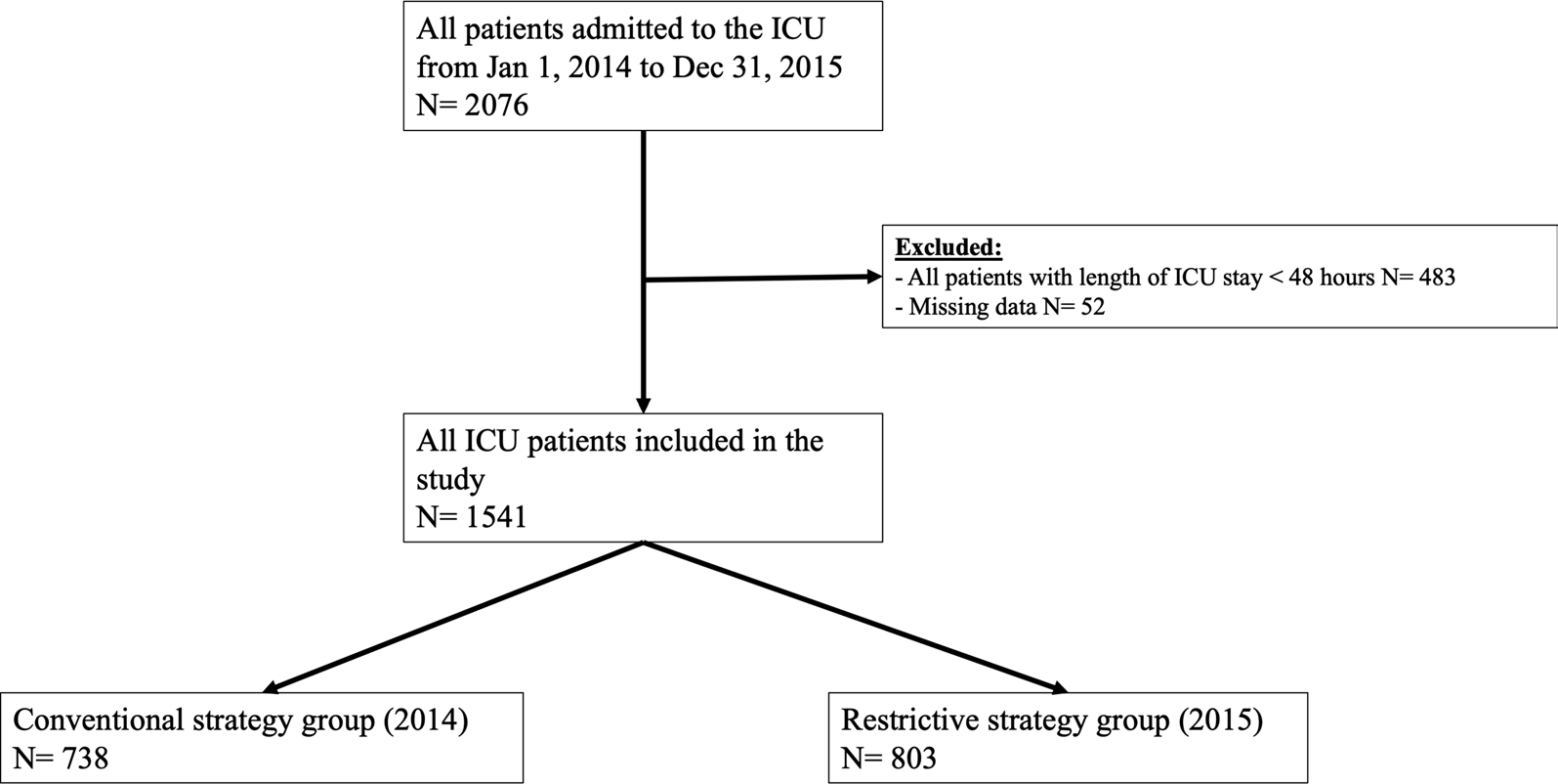
Study flowchart. *ICU* Intensive care unit

**Fig. 2:**
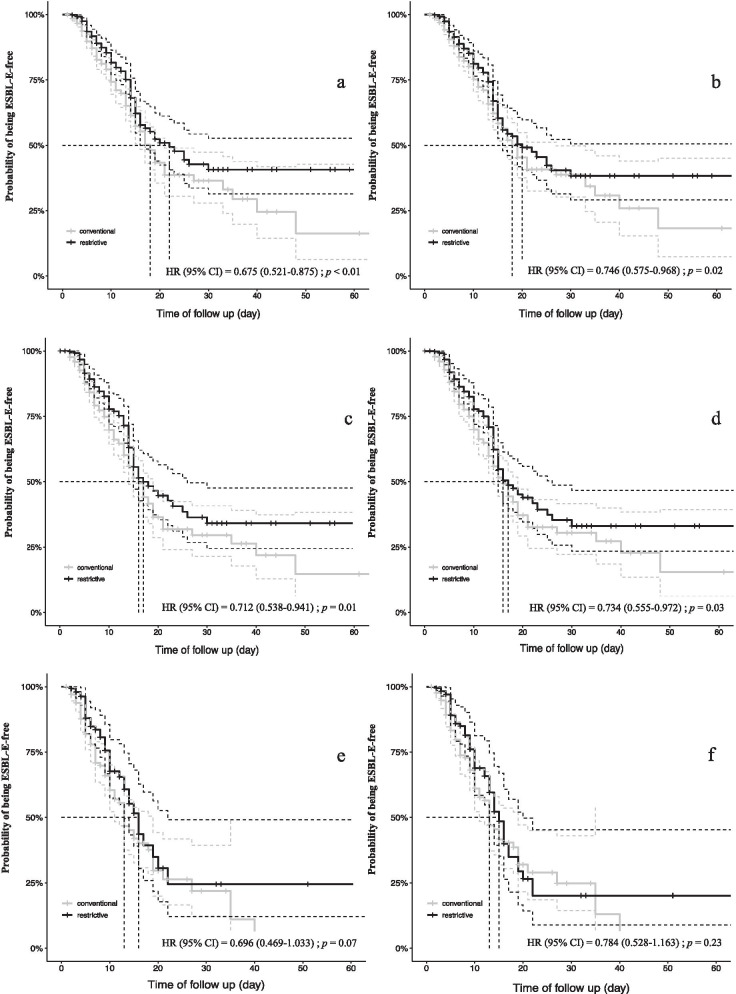
Kaplan–Meier curves for the probability of being ESBL-E-free. **a** Unweighted Kaplan–Meier survival curves obtained by the strategy used in the all-ICU patients. **b** Propensity score-weighted Kaplan–Meier survival curves obtained by the strategy used in the all-ICU patients. **c** Unweighted Kaplan–Meier survival curves obtained by the strategy used in the subgroup of patients receiving antibiotherapy. **d** Propensity score-weighted Kaplan–Meier survival curves obtained by the strategy used in the subgroup of patients receiving antibiotherapy. **e** Unweighted Kaplan–Meier survival curves obtained by the strategy used in the subgroup of patients in septic shock. **f** Propensity score-weighted Kaplan–Meier survival curves obtained by the strategy used in the subgroup of patients in septic shock. *ESBL-E*: Extended-spectrum beta-lactamase-producing *Enterobacteriaceae, HR*: hazard ratio

To balance confounding factors, a propensity score to receive the restrictive strategy was calculated using a logistic regression model including either clinically relevant or statistically significant covariates (age, Simplified Acute Physiology Score [SAPS] II, chronic renal failure, diabetes, immunosuppression, antibiotherapy prior to admission, sepsis [at admission or occurring during ICU stay], other multidrug bacteria carriage at admission, and length of ventilation > 48 h) (Fig. 1, Additional file 1). Fifty-two patients were excluded due to missing values on relevant variables. A comparison between excluded and included patients showed a significantly superior number of included patients with < 48 h ventilation (52% vs. 17.3%; p < 0.001) and sepsis at admission (33.2% vs. 5.8%; *p* < 0.001). The area under the ROC curve estimating the predictive score ability was 0.612 (95% CI 0.609–0.614) (Fig. 2, Additional file 1). In the weighted dataset, all absolute standardized differences were inferior to 5%, thus reflecting the good comparability of both groups. This score was used to calculate the inverse probability of treatment weights, assigning patients receiving a restrictive strategy a weight of 1 ÷ (propensity score) and those receiving a conventional strategy a weight of 1 ÷ (1—propensity score), with the use of stabilized weights to reduce variability. Balance among covariates was assessed in the weighted dataset using absolute standardized differences, and all results were inferior to 5%. These weights were then used to estimate the relationship between the strategy used and the median time of being ESBL-E-free in a univariate Cox proportional hazard regression weighted model. Kaplan–Meier curves of both groups from the weighted dataset were also estimated. A sensitivity analysis was performed by estimating the impact of the intervention using a multivariate Cox proportional hazard regression model adjusted on the propensity score values. All tests were conducted at a two-sided alpha risk of 5%. Analyses were performed using the 4.0.3 version of R.

## Results

### Patient characteristics and outcomes

We included 1541 patients in the study (CSP: 738; RSP; 803) (Fig. 1). Demographic characteristics before and after weighted propensity score analysis are summarized in Table 1. Baseline characteristics are provided in Table 2, Additional file 1. RSP patients had a lower SAPS II and less diagnosis of sepsis on admission. Less patients in the RSP presented with sepsis or septic shock (40.7% vs. 51.5%; *p* < 0.01), but there was no statistical difference in the proportion of patients receiving vasoactive drugs for septic shock during the two periods (20.3% vs. 24.3%; *p* = 0.06). A similar ICU mortality was observed in patients admitted for a length of stay < 48 h as safety criteria. No difference in a specific sepsis category was reported at ICU admission. However, more pulmonary infections and less intra-abdominal infections were diagnosed during ICU stay in the CSP group (Table 3, Additional file 1).

**Table 1:** Demographic characteristics of the whole cohort and subgroups before and after weighted propensity score analysis

Whole cohort	Before weighted PPS analysis		*p* value	After weighted PPS analysis		*p* value
	Conventional strategy period 2014	Restrictive strategy period 2015		Conventional strategy period 2014	Restrictive strategy period 2015	
	n = 738	n = 803		n = 1539.427	n = 1541.373	
Age, years, mean (± SD)	56.21 (18.49)	55.40 (19.32)	0.53	55.63 (18.67)	55.7 (19.25)	0.924
SAPS II, mean (± SD)	42.2 (21.08)	39.57 (22.03)	< 0.01	40.71 (20.90)	40.78 (22.2)	0.928
Diabetes	220 (29.8)	207 (25.8)	< 0.01	424.923 (27.6)	427.796 (27.8)	0.925
Chronic renal insufficiency	78 (10.6)	102 (12.7)	0.19	173.641 (11.3)	177.866 (11.5)	0.821
Immunosuppression	48 (6.5)	89 (11.1)	< 0.01	133.373 (8.7)	136.391 (8.8)	0.856
Antibiotherapy in the last 3 months	288 (39.0)	304 (37.9)	0.64	593.102 (38.5)	593.741 (38.5)	0.997
MDR bacteria carrier at ICU admission ^a^	51 (6.9)	68 (8.5)	0.25	116.09 (7.5)	118.888 (7.7)	0.857
Sepsis (at ICU admission or acquired during ICU stay)	380 (51.5)	327 (40.7)	< 0.001	706.351 (45.9)	706.214 (45.8)	0.970
Invasive mechanical ventilation > 48 h	438 (59.3)	364 (45.3)	< 0.001	802.415 (52.1)	802.174 (52.0)	0.964

Subgroup of antibiotherapy	n = 427	n = 376		n = 802.348	n = 803.804	
Age, years, mean (± SD)	57.24 (17.53)	56.11 (18.93)	0.538	56.55 (17.63)	56.65 (18.80)	0.908
SAPS II, mean (± SD)	44.94 (21.12)	44.51 (22.54)	0.688	44.40 (21.63)	44.49 (22.22)	0.935
Diabetes	136 (31.9)	95 (25.3)	0.04	233.098 (29.1)	235.304 (29.3)	0.922
Chronic renal insufficiency	51 (11.9)	48 (12.8)	0.724	96.529 (12.0)	98.767 (12.3)	0.876
Immunosuppression	32 (7.5)	53 (14.1)	< 0.01	84.614 (10.5)	84.99 (10.6)	0.985
Antibiotherapy in the last 3 months	242 (56.7)	230 (61.2)	0.197	472.742 (58.9)	474.143 (59.0)	0.978
MDR bacteria carrier at ICU admission ^a^	42 (9.8)	46 (12.2)	0.278	85.660 (10.7)	87.590 (10.9)	0.867
Sepsis (at ICU admission or acquired during ICU stay)	372 (87.1)	315 (83.8)	0.179	685.882 (85.5)	687.384 (85.5)	0.986
Invasive mechanical ventilation > 48 h	309 (72.4)	228 (60.6)	< 0.001	535.210 (66.7)	536.928 (66.8)	0.969

Subgroup of septic shock	n = 172	n = 156		n = 325.179	n = 329.259	
Age, years, mean (± SD)	60.88 (13.85)	62.91 (14.44)	0.183	61.77 (13.36)	61.92 (14.93)	0.892
SAPS II, mean (± SD)	54.69 (22.19)	54.22 (22.77)	0.980	54.73 (22.59)	54.48 (22.80)	0.884
Diabetes	64 (37.2)	48 (30.8)	0.219	113.156 (34.8)	115.746 (35.2)	0.924
Chronic renal insufficiency	24 (14.0)	24 (15.4)	0.714	48.133 (14.8)	51.222 (15.6)	0.788
Immunosuppression	17 (9.9)	31 (19.9)	0.011	46.225 (14.2)	47.780 (14.5)	0.914
Antibiotherapy in the last 3 months	95 (55.2)	111 (71.2)	0.003	200.219 (61.6)	205.274 (62.3)	0.839
MDR bacteria carrier at ICU admission ^a^	15 (8.7)	31 (19.9)	0.004	41.819 (12.9)	45.429 (13.8)	0.724
Invasive mechanical ventilation > 48 h	149 (86.6)	108 (69.2)	< 0.001	257.203 (79.1)	258.344 (78.5)	0.843

When not specified, results are n (%).

*PPS* propensity score, *SAPS II* Simplified Acute Physiology Score II; *MRSA* methicillin-resistant *Staphylococcus aureus*, *ESBL-E:* Extended-spectrum beta-lactamase-producing *Enterobacteriaceae*, *MDR* multidrug resistant, *SD* standard deviation.

^a^Multidrug-resistant bacteria including ESBL-E and MRSA.

**Table 2:** Sepsis events and antibiotherapy characteristics in the ICU during the study period

Sepsis events and antibiotherapy characteristics	Conventional strategy period 2014 n = 738	Restrictive strategy period 2015 n = 803	*p* value
**No. patients with at least one sepsis event n (%) (community or acquired)**	**380 (51.5)**	**327 (40.7)**	< 0.01
Catecholamines administered for sepsis	179 (24.3)	163 (20.3)	0.06
**No. patients receiving antibiotics n (%)**	**427 (57.9)**	**376 (46.8)**	< 0.01
No. of different antibiotics (median ± IQR)	2 [1–3]	2 [1–3]	0.55
Duration of antibiotic therapy (days, median ± IQR)	6 [4–10]	5 [3–8]	< 0.01
Antibiotic-free days until ICU discharge (days, median ± IQR)	0 [0–6]	2 [0–7]	0.03
**Antibiotics administered n (%)**			
Amoxicillin	29 (6.8)	73 (19.4)	< 0.01
Amoxicillin/clavulanic acid	115 (26.9)	65 (17.3)	< 0.01
Oxacillin	20 (4.7)	28 (7.4)	0.09
Piperacillin/tazobactam	170 (39.8)	17 (4.5)	< 0.01
Cefazolin (C1G)	1 (0.2)	13 (3.5)	< 0.01
Cefuroxime (C2G)	1 (0.2)	72 (19.1)	< 0.01
Cefotaxime/ceftriaxone (C3G)	192 (45.0)	159 (42.3)	0.44
Cefoxitin (cephamycin)	9 (2.1)	34 (9.0)	< 0.01
Ceftazidime	19 (4.4)	47 (12.5)	< 0.01
Cefepime	7 (1.6)	18 (4.8)	0.01
Carbapenem	52 (12.2)	13 (3.5)	< 0.01
Vancomycin	43 (10.1)	8 (2.1)	< 0.01
Aminoglycoside	162 (37.9)	114 (30.3)	0.02
Fluoroquinolone	13 (3.0)	45 (12.0)	< 0.01
Macrolide	63 (14.8)	55 (14.6)	0.96
Clindamycin	4 (0.9)	5 (1.3)	0.74
Metronidazole	17 (4.0)	19 (5.1)	0.46
Trimethoprim/sulfamethoxazole	17 (4.0)	26 (6.9)	0.06
**Antibiotics targeting anaerobic pathogens** ** n (%)**^a^	**279 (65.3)**	**126 (33.5)**	< 0.01

When not specified, results are n (%)

*C1G* first-generation cephalosporin, *C2G* second-generation cephalosporin, *C3G* third-generation cephalosporin, *IQR* interquartile range.

^a^Amoxicillin/clavulanic acid, piperacillin/tazobactam, carbapenem, cefoxitin, clindamycin, metronidazole.

**Table 3:** Secondary outcomes in the whole cohort and in the subgroup of patients receiving antibiotic therapy in the ICU

Secondary outcomes	Conventional strategy period 2014	Restrictive strategy period 2015	p value
**In the whole cohort n (%)**	**n = 738**	**n = 803**	
ICU-acquired ESBL-E ^a^	140 (19.0)	97 (12.1)	< 0.01
*Klebsiella pneumoniae* ESBL	124 (87.9)	81 (83.5)	0.34
*Escherichia coli* ESBL	4 (2.9)	5 (5.2)	0.49
*Enterobacter cloacae* ESBL	27 (19.3)	20 (20.6)	0.80
Others	2 (1.4)	0 (0)	0.5
ESBL-E infections	61 (8.3)	41 (5.1)	0.01
ESBL-E bacteremia	34 (4.6)	35 (4.4)	0.8
Duration of mechanical ventilation, days (median ± IQR) n = 1023	5 [3–11]	4 [3–9]	0.06
Relapse or recurrence of sepsis during ICU stay	178 (24.4)	135 (16.8)	< 0.01
All-cause ICU mortality	211 (28.6)	181 (22.5)	< 0.01
All-cause hospital mortality	253 (34.3)	222 (27.6)	< 0.01
ICU length of stay, days (median ± IQR)	6 [4–12]	5 [4–10]	< 0.01
Hospital length of stay, days (median ± IQR)	14 [7–27]	13 [6.5–23]	0.04
Patients who did not receive antibiotic therapy	311 (42.1)	427 (53.2)	< 0.01
**Patients receiving antibiotic therapy in the ICU n (%)**	**n = 427**	**n = 376**	< 0.01
ICU-acquired ESBL-E	126 (29.5)	80 (21.3)	< 0.01
ESBL-E infections	60 (14.1)	38 (10.1)	0.09
ESBL-E bacteremia	34 (8.0)	32 (8.5)	0.78
Relapse or recurrence of sepsis during ICU stay	176 (41.9)	132 (35.2)	0.05
All-cause ICU mortality	148 (34.7)	105 (27.9)	0.04
All-cause hospital mortality	246 (57.6)	255 (67.8)	< 0.01
Antibiotic-free days until ICU discharge, days mean ± SD	4.8 ± 9.5	5.7 ± 9.6	0.03
Duration of antibiotic therapy, days, mean ± SD	8.2 ± 6.8	6.7 ± 5,6	< 0.01
**Patients in septic shock n (%)**	**n = 172**	**n = 156**	0.09
ICU-acquired ESBL-E	72 (41.9)	38 (24.4)	< 0.01
ESBL-E infections	43 (25.0)	23 (14.7)	0.02
ESBL-E bacteremia	25 (14.5)	17 (10.9)	0.33
All-cause ICU mortality	103 (59.9)	73 (46.8)	0.02
All-cause hospital mortality	109 (63.4)	79 (50.6)	0.02
Antibiotic-free days until ICU discharge, days mean ± SD	5.1 ± 10.7	5.0 ± 10.3	0.77
Duration of antibiotic therapy, days, mean ± SD	10.4 ± 7.7	7.5 ± 6.8	< 0.01
**All patients with an ICU length of stay < 48 h n (%)**	**n = 242**	**n = 241**	0.3
All-cause ICU mortality	65 (26.9)	66 (27.4)	0.9
All-cause hospital mortality	76 (31.4)	71 (29.5)	0.6

When not specified, results are n (%).

*ICU* intensive care unit, *ESBL-E* extended-spectrum beta-lactamase-producing *Enterobacteriaceae*, *SD* standard deviation, *IQR* interquartile range.

^a^ Several patients acquired more than one ESBL species. This explains why the cumulative proportions of each group of patients with ESBL species are over 100%.

### Antibiotic use

During the RSP, the number of patients treated with antibiotics was significantly lower than during the CSP (46.8% vs. 57.9%, respectively; *p* < 0.01). Median duration of antibiotic treatment was shorter by one day in the RSP (5 vs. 6 days; *p* < 0.01) (Table 2). Third-generation cephalosporins (ceftriaxone, cefotaxime) were the most commonly administered antibiotics in both periods. More patients were treated with amoxicillin and cefuroxime in the RSP and less patients received amoxicillin–clavulanate. Moreover, the administration of piperacillin/tazobactam and carbapenem was significantly reduced in the RSP (4.5% vs. 39.8%, p < 0.01 and 3.5% vs. 12.5%, *p* < 0.01, respectively). Conversely, there were more patients treated with ceftazidime, cefepime and cefoxitin in the RSP compared to the CSP. Importantly, the use of antibiotics targeting anaerobes pathogens (amoxicillin/clavulanic acid, piperacillin/tazobactam, carbapenem, metronidazole, cefoxitin and clindamycin) decreased in the RSP (65.3% vs. 33.5%; *p* < 0.01) and a large number of patients did not receive any antibiotic treatment during ICU stay.

### ESBL-E acquisition in the ICU

The incidence of ICU-acquired ESBL-E was lower in the RSP compared to the CSP (12.1% vs. 19.0%; *p* < 0.01) (Table 3). The main microorganisms identified were *Klebsiella pneumoniae* (86.1%), *Enterobacter* spp*.* (19.8%) and *Escherichia coli* (3.8%). The incidence of ICU-acquired ESBL-E infection was lower in the RSP (8.3% vs. 5.1%; *p* = 0.01); ICU-acquired ESBL-E bacteremia did not differ between the study periods. No carbapenemase-producing *Enterobacteriaceae* were isolated during the study period.

The median time of being ESBL-E-free was 19 days (95% CI 17–25) in the whole cohort. In univariate analysis, the median time of being ESBL-E-free was significantly shorter in the CSP compared to the RSP with 18 days (95% CI 16–21) and 22 days (95% CI 16-NA), respectively (hazard ratio [HR], 0.675 [95% CI 0.521–0.875]; *p* < 0.01) (Fig. 2). After propensity score analysis, RSP was significantly associated with the median time of being ESBL-E-free in the ICU as a protective factor (HR, 0.746 [95% CI 0.575–0.968]; *p* = 0.02) by weighting or by adjustment (HR, 0.751 [95% CI 0.578–0.977]; *p* = 0.03) (Table 4).

**Table 4:** Multivariate analysis associated with the median time of being ESBL-E-free

Variable	Univariate analysis HR (95% CI)	*p* value	Multivariate PPS weighted analysis HR (95% CI)	*p* value	Multivariate PPS adjusted analysis HR (95% CI)	*p* value
**In the whole cohort**						
Restrictive strategy (2015)	0.675 (0.521–0.875)	< 0.01	0.746 (0.575–0.968)	0.02	0.751 (0.578–0.977)	0.03
**Patients receiving antibiotherapy**						
Restrictive strategy (2015)	0.712 (0.538–0.941)	0.01	0.734 (0.555–0.972)	0.03	0.738 (0.556–0.980)	0.03
**Patients in septic shock**						
Restrictive strategy (2015)	0.696 (0.469–1.033)	0.07	0.784 (0.528–1.163)	0.23	0.760 (0.509–1.132)	0.18

Statistical significance level < 0.05. All these variables were included to calculate the propensity score (age, SAPS II, chronic renal failure, diabetes, immunosuppression, antibiotherapy prior to admission, sepsis (at admission or occurring during ICU stay), other multidrug bacteria carriage at admission, mechanical ventilation duration > 48 h).

*ICU* intensive care unit, *CI* confidence interval, *HR* hazard ratio, *ESBL-E* extended-spectrum beta-lactamase-producing *Enterobacteriaceae*, *PPS* propensity score.

A subgroup analysis of patients receiving antibiotic therapy during ICU stay showed similar results (Tables 3 and 4; Fig. 2). In septic shock patients, the incidence of ICU-acquired ESBL-E, as well as ESBL-E infections, was lower in the RSP compared to the CSP in univariate analysis, but these results were not confirmed after weighted-propensity score analysis.

### Secondary outcomes

All-cause ICU mortality was lower in the RSP than in the CSP (22.5% vs. 28.6%, respectively; *p* < 0.01), including in the subgroups of patients receiving antibiotic therapy and in septic shock. A similar ICU mortality was observed in patients admitted for a length of stay < 48 h as safety criteria (Table 3).

## Discussion

This study provides interesting data from a restrictive antibiotic stewardship program in the ICU, with a particular emphasis on ESBL-E acquisition. An important finding was a reduction in ESBL-E acquisition with no excess in the mortality rate in the context of high prevalence in an endemic region, such as the French West Indies [18]. This finding is consistent with previous studies showing a significant reduction of the incidence of antibiotic-resistant bacteria, including in the ICU setting, following the implementation of antibiotic stewardship programs [19, 20]. The lower acquisition rate in our ICU was associated with a significant reduction of the use of broad-spectrum antibiotics, mainly piperacillin/tazobactam and carbapenem and, more generally, antibiotics targeting anaerobic microbiota. Several authors have reported the role of antibiotic therapies with activity against anaerobic microbiota, as well as the beta-lactamase inhibitor, in the acquisition of multidrug-resistant Gram-negative bacteria, including ESBL-E [5, 21]. Furthermore, preserving the microbiota against antibiotics is one of the key strategies against ESBL-E acquisition [22].

The reluctance of ICU physicians to rationalize antibiotic use is often a major limitation in stewardship programs. We were able to overcome this unwillingness in our ICU, resulting in a significant reduction in global antibiotic consumption, including broad-spectrum antibiotics, a shorter duration of therapy and an increased use of narrow-spectrum molecules. Interestingly, we observed a decrease in the consumption of carbapenems in the RSP, even though the prevalence of ESBL-E was high. The precise cause of the lower incidence of ESBL-E acquisition in the RSP is difficult to determine, but the decrease in the use of piperacillin/tazobactam may have played a role, as well as the choice of alternative antibiotics, such as cefoxitin. In the RSP, antibiotic duration was reduced by one day, although the baseline duration was already short. This is consistent with the current trend of a shorter treatment duration for infections such as VAP [23], intra-abdominal infections [24, 25] or bacteremia [26]. Discontinuation of antibiotic therapy in the case of negative microbiological cultures could also explain the short treatment duration, especially in VAP [27, 28]. Antibiotic duration is frequently longer than recommended in clinical practice [29], despite no better outcome and the likely promotion of bacterial or fungal superinfections [27]. Indeed, Daneman and colleagues showed that up to two-thirds of ICU patients with bacteremia did not receive a short course as recommended [30].

Lower antibiotic consumption during the RSP was also the result of the use of a lower threshold for the initiation of antibiotic therapy [7]. In the case of hemodynamically stable patients with suspected infection, antibiotic therapy was initiated only after clinical evidence of infection and microbiological documentation. In some patients, an alternative diagnosis was identified and antibiotic therapy was not initiated. This “conservative” strategy has been evaluated by Hranjec and colleagues in critically ill surgical patients and resulted in a higher appropriate initial antibiotic treatment, reduced duration of antibiotic treatment and a lower mortality rate [10]. Despite the contradiction of delaying the initiation of antimicrobial treatment with preexisting dogma and the promoted de-escalation strategy, several arguments support this approach [31]. First, a high proportion of patients with fever received antimicrobial therapy in the ICU, despite non-infectious disease [32]. Similarly, overdiagnosis of VAP was reported in 68% of patients, resulting in an overuse of antimicrobial therapy [33]. Second, the linear association between the timing of antibiotic therapy and mortality has been challenged [34], suggesting no benefit in an immediate start of antimicrobial drugs in less severely ill patients. Third, this position has been recently advocated by many experts [35]. Of note, the Infectious Diseases Society of America decided not to endorse the 2018 Surviving Sepsis Campaign experts and highlighted that its recommendation of prompt antibiotic therapy [36], which they regarded as “an oversimplified approach,” should be reconsidered and potentially delayed in less severely ill patients to avoid “treating some infections inadequately and others excessively” [37].

## Limitations

Our study has some limitations. First, it is a single-center observational study. Even if the medical staff, nursing teams, hygiene protocols and patient selection did not change during the study period, we acknowledge that undetermined variables may have played a role in the reported results. Nevertheless, our results regarding the implementation of the stewardship program and its feasibility could be extrapolated to other ICUs. Second, outcomes associated with the restrictive strategy cannot be addressed accurately as patients in the RSP were less severe, based on the SAPS II at admission. However, SAPS II was included in the propensity score analysis. Furthermore, the standardized mortality ratio (ratio of actual ICU mortality to SAPS II predicted mortality) was similar (0.93 and 0.95, respectively) in both periods, thus suggesting no excess in the RSP mortality rate. Third, infections may have been overdiagnosed during the CSP where the diagnosis was based on the physician’s judgment. As previously mentioned, the diagnosis of infection may be difficult in the ICU and many non-infectious diseases may present with fever or an inflammatory syndrome, especially for VAP [33]. Conversely, during the RSP, a microbiological confirmation and/or a severity signs of sepsis were required to define infection. In the data collection process, these situations were called “infection,” probably leading to some falsely reported infections during the CSP. As a suspicion of infection was often the trigger for initiating an empirical antibiotic therapy during the CSP, it is likely that an overdiagnosis of infection played a role in the broader use of antibiotics compared to the RSP where a microbiological confirmation of infection and/or a severity sign was required to initiate empirical antibiotics. Nevertheless, the proportion of patients treated with vasoactive drugs for septic shock was similar, suggesting no difference in the incidence of the most severely ill patients between groups. In addition, despite limited availability at our institution, molecular microbiological methods diagnosis, rapid diagnostic tests or biomarkers, such as procalcitonin, could contribute to make a diagnosis of sepsis at an earlier stage for severely ill patients and thus avoid delaying antibiotic therapy until standard microbiological identification was available. Fourth, the rate of initial appropriate antibiotic therapy in septic shock was not reported, including the rate of compliance to the protocol and the time to control for the infection source in the management of sepsis. The use of cefoxitin as empirical treatment for acquired non-pulmonary septic shock was based on our local epidemiology, i.e., a high prevalence of cefoxitin susceptibility to ESBL-E, a low incidence of AmpC-hyperproducing *Enterobacteriaceae* and a rare incidence of carbapenemase-producing *Enterobacteriaceae* and methicillin-resistant *Staphylococcus aureus*. Although administration of cefoxitin was appropriate when referring to antimicrobial susceptibility testing, few data support its clinical use in severe infections, other than urinary tract infection (38). To address this issue, we used a combination therapy of cefoxitin and amikacin for empirical treatment in acquired septic shock.

## Conclusions

Our results suggest that a restrictive strategy delaying the initiation of antibiotic treatment in less severely ill patients and using the narrowest spectrum has the potential to avoid the use of broad-spectrum antibiotics, particularly those targeting intestinal anaerobe microbiota. This strategy could lead to a decrease in antibiotic consumption and ESBL-E acquisition in the ICU. Appropriate randomized controlled trials are needed to evaluate this restrictive strategy to confirm these findings.

## Supplementary Information

**Additional file 1**. Additional information about the restrictive antibiotic protocol, the results and the statistical analysis (file format in .pdf). I/ Restrictive antibiotic treatment protocol from 1 January 2015 to 31 December 2015. Part 1. Initiation of antibiotic therapy. Part 2. Choice of the molecule. Part 3. Duration therapy. II/ Additional Tables and Figures. Table 1a: Prevalence of resistant bacteria in the hospital and in the ICU, 2014–2015. Table 1b: Prevalence of resistant bacteria in the hospital according to the unit and the origin of samples, 2014–2015. Table 2: Demographic characteristics, comorbidities, and diagnosis upon admission to the ICU of all included patients. Table 3: Sepsis category during the study period. Figure 1a: Absolute main differences before and after weighted adjustment in the main analysis sample. Figure 1b: Absolute main differences before and after weighted adjustment in the subgroup receiving antibiotherapy. Figure 1c: Absolute main differences before and after weighted adjustment in the subgroup in septic shock. Figure 2: ROC curve of the propensity score in the main analysis.

## Data Availability

After publication, the data will be made available upon reasonable request to the corresponding author. A proposal with a detailed description of study objectives and statistical analysis plan will be needed for evaluation of the reasonability of requests. Additional materials might also be required during the process of evaluation. De-identified participant data will be provided after approval from the corresponding author and the Guadeloupe University Hospital, Guadeloupe, French West Indies.

## Abbreviations

ESBL-E: Extended-spectrum beta-lactamase-producing *Enterobacteriaceae*
ICU: Intensive care unit
CSP: Conventional strategy period
RSP: Restrictive strategy period
ARDS: Acute respiratory distress syndrome
VAP: Ventilator-associated pneumonia
SAPS II: Simplified Acute Physiology Score II
