# Supplementary material for: Impact of a restrictive antibiotic policy on the acquisition of extended-spectrum beta-lactamase-producing Enterobacteriaceae in an endemic region: a before-and-after, propensity-matched cohort study in a Caribbean intensive care unit
Source: Crit Care. 2021 Jul 26;25:261. doi: 10.1186/s13054-021-03660-z (PMC8311634; doi:10.1186/s13054-021-03660-z)

**ADDITIONAL FILE 1**

**Impact of a restrictive antibiotic policy on the acquisition of extended-spectrum beta-lactamase-producing Enterobacteriaceae in an endemic region: a before-and-after, propensity-matched cohort study in a Caribbean intensive care unit**

Christophe Le Terrier, MD^1,2^* ; Marco Vinetti, MD,^1,3^* ; Paul Bonjean, MD^4^; Régine Richard, RN^1^; Bruno Jarrige, MD^5^; Bertrand Pons, MD^1^ ; Benjamin Madeux, MD^1^ ; Pascale Piednoir, MD^1^ ; Fanny Ardisson, MD^1^; Elain Elie, MD^1^ ; Frédéric Martino, MD^1^ ; Marc Valette, MD^1^; Edouard Ollier, MD, PhD^4^ ; Sébastien Breurec, MD, PhD^6,7,8,9^; Michel Carles,MD, PhD^1,7^; Guillaume Thiéry, MD^10,11^

* Contributed equally to this work

^1^ Division of Intensive Care, University Hospital of Guadeloupe, Pointe-à-Pitre/Les Abymes, French West Indies, France

^2^ Division of Intensive Care, Geneva University Hospitals, Geneva, Switzerland

^3^ Division of Intensive Care, Saint-Pierre Clinic, Ottignies, Belgium

^4^Division of Clinical Epidemiology, University Hospital of Saint-Etienne, Saint-Etienne, France

^5^ Division of Hospital Infection Control, University Hospital of Guadeloupe, Pointe-à-Pitre/Les Abymes, French West Indies, France

^6^ Laboratory of Clinical Microbiology, Faculty of Medecine Hyacinthe Bastaraud, University of Antilles, Pointe-à-Pitre, French West Indies, France

^7^ Faculty of Medecine Hyacinthe Bastaraud, University of Antilles, Pointe-à-Pitre, French West Indies, France

^8^ INSERM Center for Clinical Investigation 1424, Pointe-à-Pitre/Les Abymes, French West Indies, France

^9^ Transmission, Reservoir and Diversity of Pathogens Unit, Institut Pasteur de Guadeloupe, Pointe-à-Pitre, French West Indies, France

^10^ Division of Intensive Care, University Hospital of Saint-Etienne, Saint-Etienne, France

^11^ University Jean Monnet, Saint-Etienne, France

**Keywords:** intensive care unit, Caribbean, extended-spectrum beta-lactamase (ESBL)–producing Enterobacteriaceae, antimicrobial resistance, antibiotic stewardship, Intestinal microbiota, ESBL-E colonization.

**Running title**: *Antimicrobial stewardship and ESBL*

**Submitted to:** *Critical Care*

**Word count**: Abstract: 332; manuscript 3439; 4 Tables; 2 Figures; 1 Additional File

**Corresponding author:**

Christophe Le Terrier, MD

Division of Intensive Care

Geneva University Hospitals

4 Rue Gabrielle-Perret-Gentil

1211 Geneva 14, Switzerland

Tel: +41 775 53 27 41

E-mail: [leterrier.icu@gmail.com](mailto:leterrier.icu@gmail.com)

ORCID: 0000-0002-5455-5576

**Alternative corresponding author**:

Guillaume Thiéry, MD

Division of Intensive Care

University Hospital of Saint-Etienne

Avenue Albert Raimond

42270 Saint-Priest-en-Jarez / France

Tel: +33 4 77 12 78 62

E-mail: [guillaume.thiery@chu-st-etienne.fr](mailto:guillaume.thiery@chu-st-etienne.fr)

**Contents**

**I/ Restrictive antibiotic treatment protocol from 1 January 2015 to 31 December 2015**

**Part 1**. Initiation of antibiotic therapy 4

**Part 2.** Choice of the molecule 5

**Part 3.** Duration therapy 6

**II/ Additional Tables and Figures**

**Table 1** **a**. Prevalence of resistant bacteria in the hospital and in the ICU, 2014-2015 7

**b**. Prevalence of resistant bacteria in the hospital according to the unit and the origin

of samples, 2014-2015 7

**Table 2** Demographic characteristics, comorbidities, and diagnosis upon admission to the ICU of all included patients 8

**Table 3** Sepsis category during the study period 9

**Figure 1** **a.** Absolute main differences before and after weighted adjustment in the main analysis sample 10

**b.** Absolute main differences before and after weighted adjustment in the subgroup receiving antibiotherapy 10

**c.** Absolute main differences before and after weighted adjustment in the subgroup

in septic shock. 11

**Figure 2** ROC curve of the propensity score in the main analysis 11

**II/ Restrictive antibiotic treatment protocol from 1 January 2015 to 31 December 2015**

**Part 1. Initiation of antibiotic therapy**

**Stable patient** (no septic shock, no severe acute respiratory distress syndrome [ARDS], no bacterial meningitis) with suspicion of clinical sepsis:

- Collection of a complete bacteriological chart (blood cultures, urine, sputum, puncture of any other suspect liquid);
- Abstention of antibiotic treatment until microbiological evidence of infection obtained.

*Lung: 10^5^ CFU/ml in quantitative endotracheal suction specimen or 10^3^ CFU/ml in distal pulmonary samples.*

*Urinary tract: 10^5^ CFU/ml in urinary samples with significant leukocyturia (white cell count >10^4^/ml).*

*Abdominal: Any isolation in a sterile liquid (ascitis/peritoneal fluid).*

*Blood samples: Any isolation not considered contaminating (e.g., coagulase-negative staphylococci).*

*Any cavity/sterile space (pleura, joints, cerebrospinal fluid, etc.): Any isolation.*

**Unstable patient** (in septic shock, severe ARDS or suspicion of bacterial meningitis):

- Collection of a complete bacteriological chart (blood cultures, urine, sputum, puncture of any suspicious liquid).
- After sampling, immediate initiation of a combination therapy with a dose of aminoglycoside (except for treatment of an abscess or anaerobic infection).

**Part 2. Choice of the molecule**

**General rules**

• No empirical use of carbapenem or piperacillin/tazobactam (to be used only in the case of documentation with an antibiogram leaving no other choice).

• As narrow a spectrum as possible.

• No empirical coverage of subdiaphragmatic anaerobes (unless clearly indicated in management, e.g., colonic perforation).

• No empirical coverage of *Pseudomonas aeruginosa*, unless clearly indicated in management, e.g., acute chronic obstructive pulmonary disease colonized with *P. aeruginosa* or late ventilator-associated pneumonia (VAP).

• Monotherapy in definitive antibiotic treatment, except endocarditis.

• Use of single-dose aminoglycoside for spectrum broadening and sparing of broad-spectrum beta-lactams while waiting for microbiological data.

***Protocolized specific empirical treatments***

- Cefuroxime (+ metronidazole if submesocolic location) for community peritonitis
- Cefuroxime for community biliary peritonitis
- Cefoxitin or temocillin +/- metronidazole for nosocomial (tertiary) peritonitis
- Cefotaxime + spyramycin for community-acquired pneumonia
- Cefazolin for dilapidated wounds with open fractures and for facial smashing
- Cefuroxime or ciprofloxacin for urinary tract infections
- Amoxicillin for suspicion of leptospirosis
- Cefuroxime for early VAP (<5 days)
- Ceftazidime plus cloxacillin for late VAP (> 5 days)

**Part 3. Duration therapy**

**Short and fixed** duration of all antibiotic therapies, other than those defined in the long-term antibiotic therapy group (see below).

**• 5 days** for community-acquired pneumonia;

**• 4 days** for secondary peritonitis (from the surgical intervention)

**• 24 h** for proximal digestive perforations operated within 24 hours

**• <24 h** for traumatic digestive perforations (any kind) operated within 12 hours

**• 1-3 days** for dilapidated wounds with open fractures

• **24 h** for facial smears (with sinus fracture)

**• 7 days** for coagulase-negative staphylococci bacteremia

**• 14 days** for *Staphylococcus aureus* bacteremia

• **5 days** for leptospirosis

**• 7 days** for pyelonephritis

**• 7-21 days** for bacterial meningitis

- **7 days** if *Neisseria meningitidis* or *Haemophilus influenzae*
- **10 days** if *Streptococcus pneumonia*
- **14-21 days** for other etiologies, depending on the germ

**• 7 days** for VAP

**• 7 days** maximum for any other infection.

**Long-term therapies (≥14 days),** in collaboration with the infectiology team

• Osteitis and severe skin and soft tissue infections

• Endocarditis

• Spondylodiscitis

• Empyema and pulmonary abscess

• Deep abscess undrained and/or resistant to antibiotic therapy

• Complicated infected thrombophlebitis

**II/ Additional Tables and Figures**

| **Resistance rates within species in diagnostic samples *** | | **Hospital** | | **ICU** | |
| --- | --- | --- | --- | --- | --- |
|  |  | **2014** | **2015** | **2014** | **2015** |
| **ESBL-E (%)** | | **13** | **11.8** | **37.6** | **28.4** |
|  | *Klebsiella pneumoniae* | 33.3 | 28.6 | 59.4 | 52.2 |
|  | *Escherichia coli* | 3.5 | 3.4 | 3.8 | 9.1 |
|  | *Enterobacter cloacae* | 22 | 21 | 42.9 | 31.8 |
|  | *Klebsiella aerogenes* | 9 | 11 | NA | NA |
|  | Others | 1 | 1 | NA | NA |
| **Carbapenemase-producing *Enterobacteriaceae^a^* (%)** | | **0** | **0.03** | **0** | **0** |
| **MRSA (%)** | | **8.9** | **7** | **13.8** | **3.6** |
| **GRE (%)** | | **0** | **0** | **0** | **0** |
| **Resistant-ceftazidime *Acinetobacter baumanii* (%*)*** | | **2** | **2** | **8.3** | **NA** |
| **Resistant-imipenem *Acinetobacter baumanii* (%*)*** | | **2** | **1** | **8.3** | **NA** |
| **Resistant-ceftazidime *Pseudomonas aeruginosa* (%)** | | **6.2** | **10.9** | **10.4** | **7.9** |
| **Resistant-imipenem *Pseudomonas aeruginosa* (%)** | | **11.2** | **7.2** | **23.0** | **10.5** |

**Table 1a**. Prevalence of resistant bacteria in the hospital and in the ICU, 2014-2015

**b**. Prevalence of resistant bacteria in the hospital according to the unit and the origin of samples, 2014-2015.

| **Resistance rates within species in diagnostic samples *** | | **Total** | | **Urinary** | | **Catheter** | | **Blood** | | **Pulmonary** | |
| --- | --- | --- | --- | --- | --- | --- | --- | --- | --- | --- | --- |
|  |  | **2014** | **2015** | **2014** | **2015** | **2014** | **2015** | **2014** | **2015** | **2014** | **2015** |
| **ESBL-E in the hospital (%)** | | **13** | **11.8** | **12.5** | **14** | **50** | **33** | **19.1** | **21.3** | **21.8** | **21.8** |
|  | *ICU* | 33.3 | 28.6 | 33 | 30 | 56.3 | 38 | 45 | 40 | 24 | 17.5 |
|  | *Medicine unit* | 19.7 | 20.9 | 18.9 | 18.9 | 36.8 | 36.8 | 19.1 | 27 | 29.4 | 16.6 |
|  | *Surgical unit* | 10.8 | 12 | 14.6 | 21 | 20.7 | 25 | 25 | 16.2 | 0 | 28.5 |
| **ESBL-producing *K. pneumoniae* in the hospital (%)** | | **32** | **28.5** | **31** | **32** | **70** | **54** | **42.5** | **36** | **43.3** | **27** |
|  | *ICU* | 59 | 52 | 59 | 60 | 73 | 58 | 71 | 61 | 43.7 | 30 |
|  | *Medicine unit* | 38.5 | 38 | 39 | 44.9 | 60 | 37 | 32.5 | 35.4 | 66 | 14 |
|  | *Surgical unit* | 23.9 | 31 | 26 | 37 | 50 | 50 | 62.8 | 33 | 0 | 50 |

*Rate of resistance strains within species in only samples for diagnostic purposes. Ecological sampling and duplicates were excluded.

ESBL-E: extended-spectrum beta-lactamase–producing *Enterobacteriaceae;* MRSA: methicillin-resistant *Staphylococcus aureus*; GRE: glycopeptide-resistant enterococci; NA: Missed data.

^a^ Two strains were detected in the hospital in carriage in 2014: one strain of NDM-producing *K. pneumoniae* and one strain OXA-48-producing *E. coli*; in 2015, one strain was detected in the hospital in carriage of KPC-2-producing *K. pneumoniae.*

**Table 2.** Demographic characteristics, comorbidities, and diagnosis upon admission to the ICU of all included patients

| **Patient characteristics** | | **Conventional strategy period**  **2014**  **n=738** | **Restrictive strategy period**  **2015**  **n=803** | ***p* value** |
| --- | --- | --- | --- | --- |
| Age, years, median (IQR) | | 59 (45-69) | 58 (42-70) | 0.53 |
| Male (%) | | 64.0 | 63.1 | 0.74 |
| SAPS II, median (IQR) | | 40 (26-55) | 37 (24-53) | <0.01 |
| **Comorbidities before ICU admission n (%)** | |  |  |  |
|  | Hypertension | 284 (38.5) | 349 (43.5) | 0.05 |
|  | Diabetes | 220 (29.8) | 207 (25.8) | <0.01 |
|  | Chronic renal insufficiency | 78 (10.6) | 102 (12.7) | 0.19 |
|  | Cardiac disease | 73 (9.9) | 67 (8.3) | 0.29 |
|  | Immunosuppression | 48 (6.5) | 89 (11.1) | <0.01 |
|  | Malignancy | 67 (9.1) | 62 (7.7) | 0.34 |
|  | Obesity ^a^ | 52 (7.0) | 65 (8.1) | 0.44 |
|  | Hospitalization in the last year | 144 (19.5) | 205 (25.5) | <0.01 |
|  | Antibiotherapy in the last 3 months | 288 (39.0) | 304 (37.9) | 0.64 |
|  | ESBL-E carrier at ICU admission | 34 (4.6) | 35 (4.4) | 0.09 |
| **Diagnosis on admission to the ICU^b^ n (%)** | |  |  | <0.01 |
|  | Sepsis | 237 (32.1) | 211 (26.3) |  |
|  | Acute renal failure | 20 (2.7) | 30 (3.7) |  |
|  | Acute respiratory failure | 59 (8.0) | 52 (6.5) |  |
|  | Neurological failure | 115 (15.6) | 98 (12.2) |  |
|  | Polytrauma | 93 (12.6) | 137 (17.1) |  |
|  | Acute heart failure | 38 (5.1) | 78 (9.7) |  |
|  | Surgical emergency | 56 (7.6) | 46 (5.7) |  |
|  | Sickle cell disease | 11 (1.5) | 25 (3.1) |  |
|  | Suicide attempt | 22 (3.0) | 27 (3.4) |  |
|  | others | 87 (11.8) | 99 (12.3) |  |

^a^ Obesity defined by a body mass index >30

^b^Main diagnosis made at admission

# When not specified, results are n (%).

SAPS II: Simplified Acute Physiology Score II; ICU: intensive care unit; ESBL-E: extended-spectrum beta-lactamase–producing Enterobacteriacea*e;* SD: standard deviation; IQR: interquartile range.

**Table 3** Sepsis category on ICU admission and ICU-acquired during the study period

| **Sepsis category** | | **Conventional strategy period**  **2014**  **n=738** | **Restrictive strategy period**  **2015**  **n=803** | ***p* value** |
| --- | --- | --- | --- | --- |
| **On ICU admission^a^ n (%)** | | **n= 237 (32.1)** | **n= 211 (26.3)** | **<0.01** |
|  | Leptospirosis | 10 (1.4) | 16 (2.0) | 0.33 |
|  | Pulmonary infection | 111 (40.8) | 83 (34.9) | 0.17 |
|  | Urinary infection | 34 (12.5) | 44 (18.5) | 0.06 |
|  | Cutaneous infection | 8 (2.9) | 13 (5.5) | 0.15 |
|  | Catheter-related bloodstream infection | 5 (1.8) | 9 (3.8) | 0.18 |
|  | Intraabdominal infection | 58 (21.3) | 55 (23.1) | 0.63 |
|  | Endocarditis | 7 (2.6) | 7 (2.9) | 0.80 |
|  | Central nervous system infection | 23 (8.5) | 13 (5.5) | 0.19 |
|  | Bone infection | 3 (1.1) | 3 (1.3) | 1 |
|  | Ear, nose and throat infection | 3 (1.1) | 9 (3.8) | 0.05 |
|  | Others | 28 (10.3) | 18 (7.6) | 0.28 |
| **Acquired during ICU length of stay^a^ n (%)** | | **n= 178 (24.4)** | **n= 135 (16.8)** | **<0.01** |
|  | Pulmonary infection | 108 (61.0) | 61 (44.2) | <0.01 |
|  | Urinary infection | 24 (13.6) | 22 (15.9) | 0.55 |
|  | Cutaneous infection | 4 (2.3) | 6 (4.3) | 0.34 |
|  | Catheter-related bloodstream infection | 24 (13.6) | 20 (14.5) | 0.81 |
|  | Intraabdominal infection | 10 (5.6) | 32 (23.2) | <0.01 |
|  | Endocarditis | 2 (1.1) | 2 (1.4) | 1 |
|  | Central nervous system infection | 11 (6.2) | 3 (2.2) | 0.08 |
|  | Bone infection | 0 (0) | 3 (2.2) | 0.08 |
|  | Ear, nose and throat infection | 2 (1.1) | 2 (1.4) | 0.80 |
|  | Others | 28 (15.8) | 16 (11.6) | 0.70 |

^a^ Multiple diagnoses were possible for the same patient.

# When not specified, results are n (%).

SAPS II: Simplified Acute Physiology Score II; ICU: intensive care unit; ESBL-E: extended-spectrum beta-lactamase–producing Enterobacteriacea*e;* SD: standard deviation

**Figure 1a.** Absolute main differences before and after weighted adjustment in the main analysis sample.

**b.** Absolute main differences before and after weighted adjustment in the subgroup receiving antibiotherapy. **c.** Absolute main differences before and after weighted adjustment in the subgroup in septic shock.


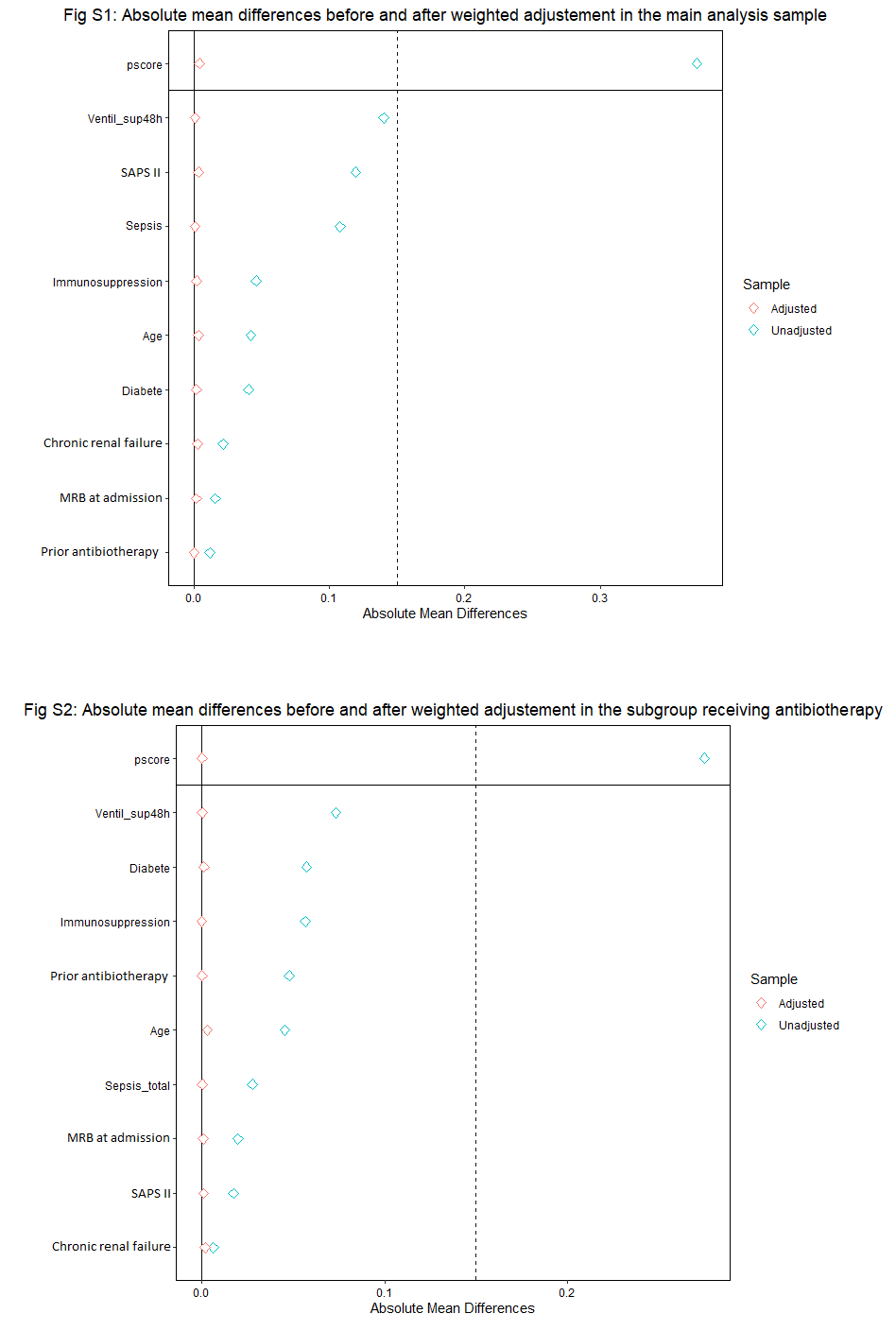

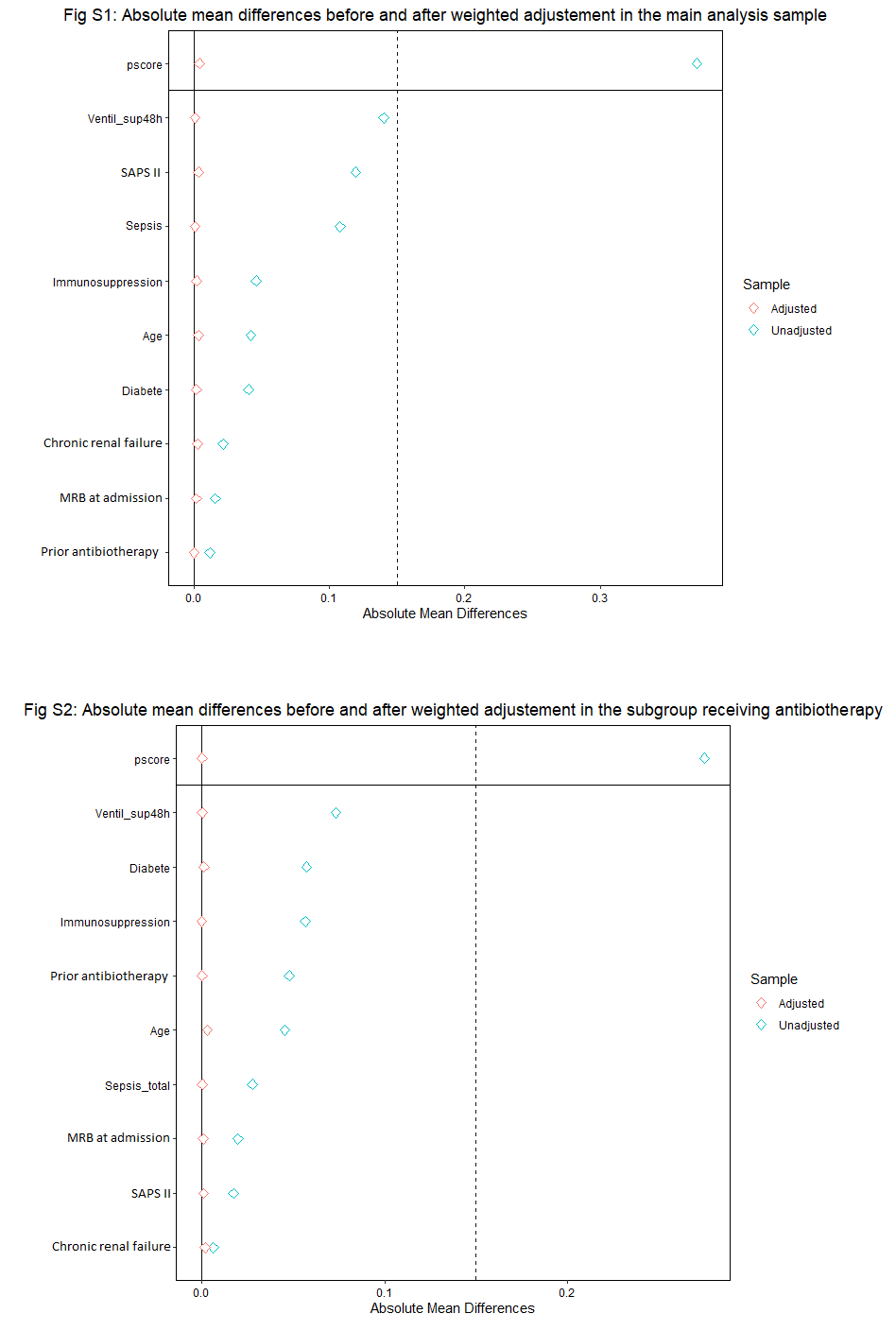


a

b


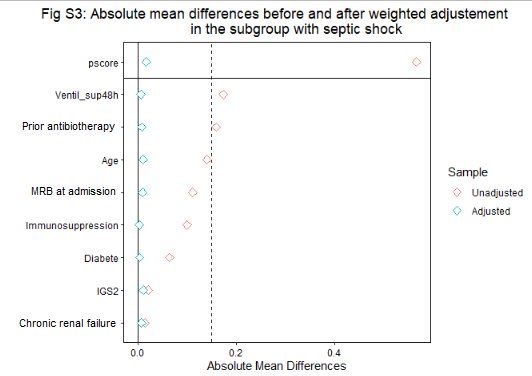


c

**Figure 2** ROC curve of the propensity score in the main analysis.


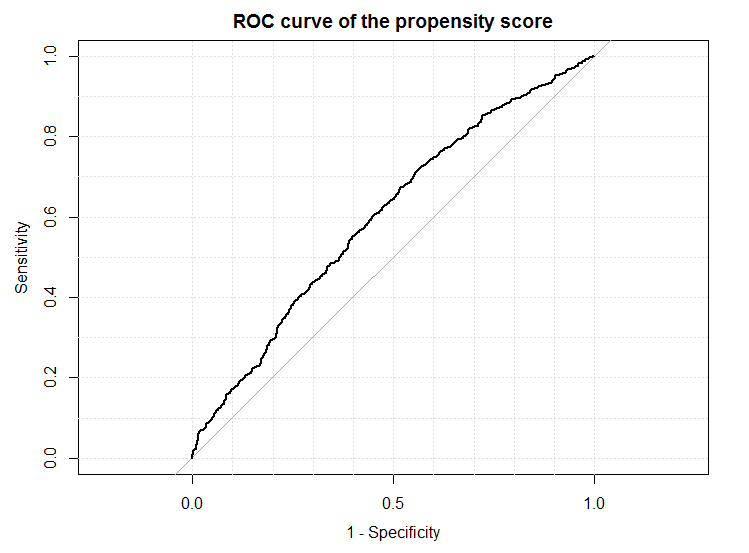

Supplement: Supplementary file 1 — Additional file 1. Additional information about the restrictive antibiotic protocol, the results and the statistical analysis (file format in .pdf). I/ Restrictive antibiotic treatment protocol from 1 January 2015 to 31 December 2015. Part 1. Initiation of antibiotic therapy. Part 2. Choice of the molecule. Part 3. Duration therapy. II/ Additional Tables and Figures. Table 1a: Prevalence of resistant bacteria in the hospital and in the ICU, 2014–2015. Table 1b: Prevalence of resistant bacteria in the hospital according to the unit and the origin of samples, 2014–2015. Table 2: Demographic characteristics, comorbidities, and diagnosis upon admission to the ICU of all included patients. Table 3: Sepsis category during the study period. Figure 1a: Absolute main differences before and after weighted adjustment in the main analysis sample. Figure 1b: Absolute main differences before and after weighted adjustment in the subgroup receiving antibiotherapy. Figure 1c: Absolute main differences before and after weighted adjustment in the subgroup in septic shock. Figure 2: ROC curve of the propensity score in the main analysis. [file 13054_2021_3660_MOESM1_ESM.docx]
